# Metabolic engineering of a reduced-genome strain of *Escherichia coli *for L-threonine production

**DOI:** 10.1186/1475-2859-8-2

**Published:** 2009-01-07

**Authors:** Jun Hyoung Lee, Bong Hyun Sung, Mi Sun Kim, Frederick R Blattner, Byoung Hoon Yoon, Jung Hoe Kim, Sun Chang Kim

**Affiliations:** 1 Department of Biological Sciences, Korea Advanced Institute of Science and Technology, Daejeon 305-701, Korea; 2 Biomass Team, Korea Institute of Energy Research, Daejeon 305-343, Korea; 3 Department of Genetics, University of Wisconsin, Madison, Wisconsin 53706, USA; 4 Scarab Genomics, Madison, Wisconsin 53713, USA

## Abstract

**Background:**

Deletion of large blocks of nonessential genes that are not needed for metabolic pathways of interest can reduce the production of unwanted by-products, increase genome stability, and streamline metabolism without physiological compromise. Researchers have recently constructed a reduced-genome *Escherichia coli *strain MDS42 that lacks 14.3% of its chromosome.

**Results:**

Here we describe the reengineering of the MDS42 genome to increase the production of the essential amino acid L-threonine. To this end, we over-expressed a feedback-resistant threonine operon (thrA*BC), deleted the genes that encode threonine dehydrogenase (*tdh*) and threonine transporters (*tdcC *and *sstT*), and introduced a mutant threonine exporter (*rhtA23*) in MDS42. The resulting strain, MDS-205, shows an ~83% increase in L-threonine production when cells are grown by flask fermentation, compared to a wild-type *E. coli *strain MG1655 engineered with the same threonine-specific modifications described above. And transcriptional analysis revealed the effect of the deletion of non-essential genes on the central metabolism and threonine pathways in MDS-205.

**Conclusion:**

This result demonstrates that the elimination of genes unnecessary for cell growth can increase the productivity of an industrial strain, most likely by reducing the metabolic burden and improving the metabolic efficiency of cells.

## Background

The vast increase in annotated genome information and high-throughput technologies has enabled a systematic improvement of industrial microbes through genome engineering. Restructuring of microbial genomes has been shown to have several advantages over conventional approaches for strain improvement [1-5]. Restructured genomes with the desired functionalities have served as customized industrial strains that display (i) streamlined metabolic pathways for the production of selected biomaterials, (ii) a reduced production of unwanted by-products, and (iii) increased genome stability [6-9].

*Escherichia coli *is the most commonly used microbe for both laboratory research and industrial-scale production of metabolites, such as amino acids and proteins, for therapeutic or commercial uses [10-14]. Because *E. coli *has adapted to a lifestyle that includes residence in animal intestines with frequent exposure to aqueous and soil environments, many of the genes in its genome are unnecessary for growth in the relatively simple environment within industrial fermentors. Further, the complete genome sequence of *E. coli *has revealed numerous genes whose products have no known functions and genetic materials that may have been acquired from other organisms in the recent past [15]. In an effort to improve *E. coli *as an industrial host, many researchers have deleted or added limited numbers of selected genes to the genome or modified plasmids to complement the existing genome [16-24]. These efforts have helped researchers make significant progress in improving *E. coli *as a production host, but have not addressed the productivity problems caused by the numerous *E. coli *genes with potentially detrimental functions.

Recently, an *E. coli *genome was reduced by the precise deletion of nonessential genes and other DNA sequences-including all known recombinogenic and mobile DNA and cryptic virulence genes-to construct a genetically stable strain that displays robust metabolic performance [5]. The resulting strain, *E. coli *MDS42, has a chromosome that is 14.3% smaller than that of its parental *E. coli *strain MG1655. MDS42 shows robust growth under normal laboratory conditions and even better growth in high-cell density fermentations, as well as increased transformation efficiency relative to MG1655 [5]. Therefore, the elimination of unnecessary genes and sequences from an *E. coli *genome appears to have produced a stable reduced-genome strain without physiological compromise. Furthermore, the deletion of all insertion sequence (IS) elements from the genome means that the strain is free of IS-mediated mutagenesis and genomic rearrangements.

In this study, we report the results of a reengineering of MDS42 to increase production of the amino acid L-threonine, which is essential for growth and maintenance of commercial livestock [25]. This reengineering included the overexpression of a feedback-resistant threonine operon (thrA*BC) under the control of a recombinant *Tac *promoter, deletion of the genes that encode threonine dehydrogenase (*tdh*) and threonine uptake proteins (*tdcC *and *sstT*), and introduction of a mutant threonine exporter gene (*rhtA23*). The reengineered strain, called MDS-205, shows an ~83% increase in threonine production by flask fermentation relative to the wild-type *E. coli *strain MG1655 that had been engineered to carry the same threonine-specific modifications. Minimization of an *E. coli *genome by the elimination of genes unnecessary for growth increases the productivity of the strain by reducing the metabolic burden caused by maintenance and expression of unnecessary genes and improving the metabolic efficiency of the cell.

## Methods

### Bacterial strains, plasmids, enzymes, and chemicals

The bacterial strains used in this work are listed in Table 1. Plasmid pKD46 was obtained from B. L. Wanner [26], pST76-ASceP from G. Posfai [27], and pCSI from S. C. Kim [28]. All enzymes were purchased from New England Biolabs (Beverly, MA, USA) except *Taq *polymerase, which was from Takara Bio Inc. (Shiga, Japan). All antibiotics and chemicals were from Sigma-Aldrich (St. Louis, MO, USA). Ampicillin, chloramphenicol, and kanamycin were used at concentrations of 50, 17, and 25 μg/ml, respectively. All the primers used in this work are listed in Additional file 1.

**Table 1:** *E. coli *strains used in this study

**Strain name**	**Description**	**Source or reference**
MG1655	*λ *^-^*F*^-^*ilvG rfb-50 rph-1*	Blattner *et al*. (1997)
MG-102	*MG1655 P*_*Tac*_-thrA*BC ΔlacI	This study
MG-103	*MG1655 P*_*Tac*_-thrA*BC ΔlacI Δtdh	This study
MG-104	*MG1655 P*_*Tac*_-thrA*BC ΔlacI Δtdh ΔtdcC::*rhtA23*	This study
MG-105	*MG1655 P*_*Tac*_-thrA*BC ΔlacI Δtdh ΔtdcC::*rhtA23 ΔsstT*::*rhtA23*	This study
MDS42	*Reduced genome strain*	Posfai *et al*. (2006)
MDS-202	*MDS42 P*_*Tac*_-thrA*BC ΔlacI	This study
MDS-203	*MDS42 P*_*Tac*_-thrA*BC ΔlacI Δtdh	This study
MDS-204	*MDS42 P*_*Tac*_-thrA*BC ΔlacI Δtdh ΔtdcC::*rhtA23*	This study
MDS-205	*MDS42 P*_*Tac*_-thrA*BC ΔlacI Δtdh ΔtdcC::*rhtA23 ΔsstT*::*rhtA23*	This study
MG1655 *thrB::Tn5*	*MG1655 thrB*::*Tn5*	Yu *et al*. (2002)
N99 rhtA23	*W3350 rpsL rhtA23 thr*::*Tn10*	Livshits *et al*. (2003)
*E. coli *ATCC 21277	*K-12 SupE relA*^+^*Km*^R^-*P*_*TAC*_-thrA*BC ilvA422	Shiio *et al*. (1971)

### Construction of threonine-producing *E. coli *strains

**Figure 1:**
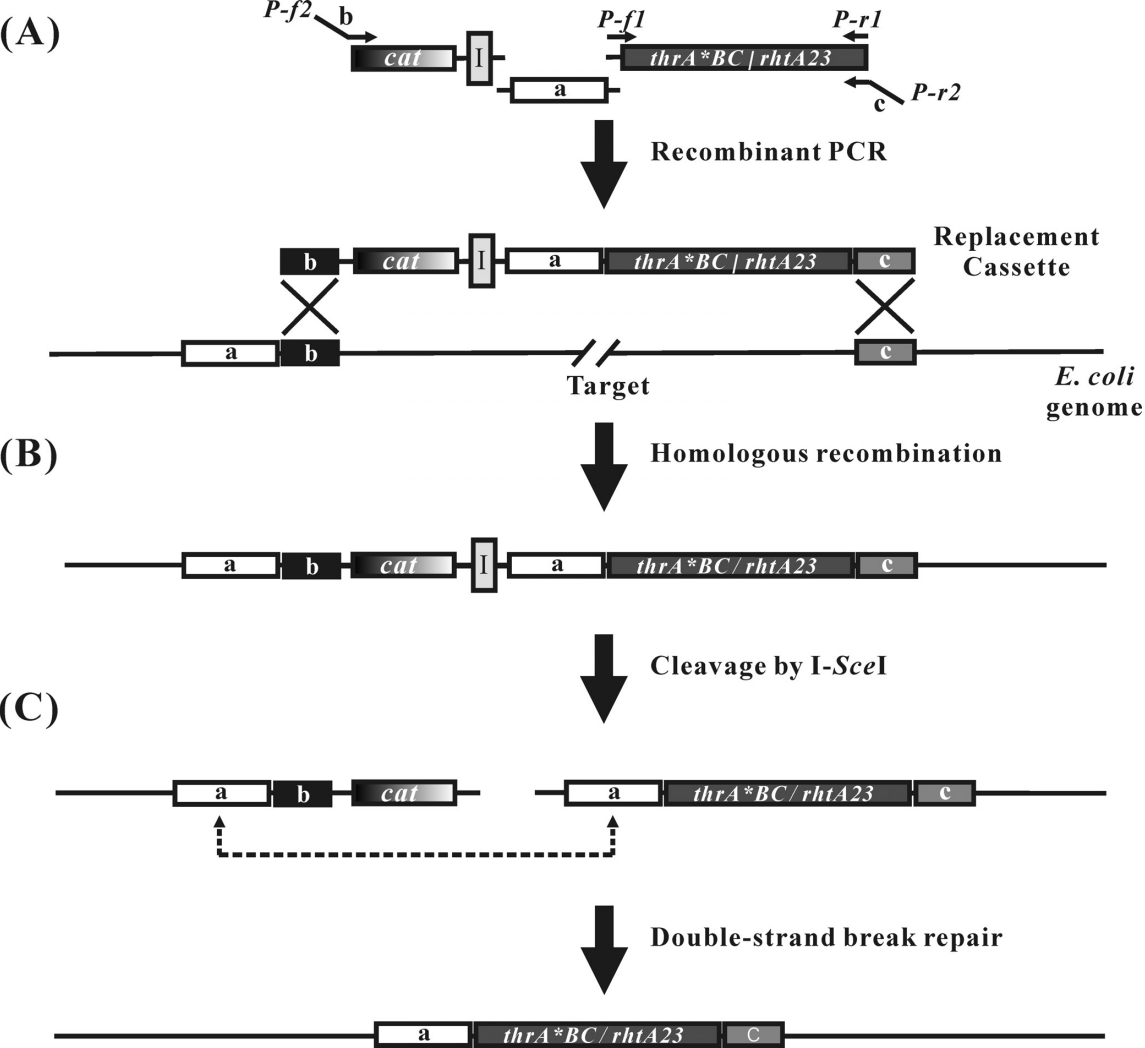
**Markerless replacement of a target**. **(A) **A linear DNA cassette containing a positive selection marker (*cat*), an I-*Sce*I recognition site (I), a gene to be replaced (thrA*BC or *rhtA23*), and three homology arms (represented as a, b, and c) were amplified by recombinant PCR (refer to Materials and Methods). PCR primers are labeled with lower case, italicized letters (*P-f1*, *P-r1*, *P-f2*, and *P-r2*) and arrows. **(B) **The target of the *E. coli *genome was replaced by the constructed DNA cassette, and recombinants were selected on LB plates containing chloramphenicol. **(C) **The *cat *gene introduced was excised from the selected recombinants by double-strand break repair mediated by I-*Sce*I cleavage.

To study the effect of minimization of an *E. coli *genome on the production of L-threonine, the reduced-genome strain MDS42 [5] and wild-type strain MG1655 were engineered to produce L-threonine. First, to release the feedback inhibition on aspartokinase I and homoserine dehydrogenase I that are encoded by the *thrA *gene, and the transcriptional attenuation of the *thrABC *operon by intracellular threonine [29], a feedback-resistant thrA*BC operon under the control of a *Tac *promoter [30] was introduced to replace the wild-type *thrABC *operon in the MG1655 and MDS42 genomes (Fig. 1). The feedback-resistant thrA*BC operon (3.5 kb) was amplified by the polymerase chain reaction (PCR) from *E. coli *ATCC 21277 genomic DNA with the following forward (P-f1) and reverse (P-r1) primers (see Fig. 1). A 1.2-kb fragment containing a chloramphenicol resistant gene (*cat*) and an I-*Sce*I recognition site was also amplified by PCR from plasmid pCSI [28]. Finally, a 0.5-kb homology fragment that sits to the left of the *thrABC *operon (Fig. 1a) was PCR-amplified from the chromosomal DNA of MDS42 and MG1655. This amplified 0.5-kb fragment contained a short, 20-base pair (bp) flanking sequence on each side: the 3' end of the 0.5-kb fragment overlapped with the 5' end of the 3.5-kb fragment and the 5' end overlapped with the 3' end of the 1.2-kb fragment described above. This 0.5-kb fragment and 2 PCR fragments above (that is, the 3.5-kb PCR fragment, which contained the feedback-resistant thrA*BC operon under the control of a *Tac *promoter, and the 1.2-kb fragment, which contained a *cat *gene and an I-*Sce*I recognition site) were combined by recombinant PCR using the following forward (P-f2) and reverse (P-r2) primers (see Fig. 1A). The constructed 5.3-kb DNA cassette was electrotransformed into MG1655 and MDS42, both of which harbored pKD46 expressing λ-Red genes (*γ*, *β*, and *exo*). The recombinants were selected on chloramphenicol-containing LB plates, and then the helper plasmid pKD46 was cured by growing the selected recombinants at 42°C. The *cat *gene that was introduced as described above was then excised from the constructed recombinant strains by double-strand break (DSB) repair mediated by the I-*Sce*I endonuclease expressed from pST76-ASceP, which generated strain MG-101 from MG1655 and strain MDS-201 from MDS42. To constitutively express the feedback-resistant thrA*BC operon under the control of a *Tac *promoter, the *lacI *gene was deleted from the MG-101 and MDS-201 genomes by the markerless deletion method [28], producing MG-102 and MDS-202, respectively. To prevent the degradation of L-threonine produced inside the cells, the threonine dehydrogenase gene (*tdh*) was deleted from the MG-102 and MDS-202 genomes by the markerless deletion method, generating strains MG-103 and MDS-203, respectively. To further increase threonine production, two threonine uptake genes, *tdcC *and *sstT*, were sequentially replaced by the mutant threonine exporter gene (*rhtA23*) (see Fig. 1), whose expression level is 10-fold higher than that of the wild-type *rhtA *gene [31]. These manipulations yielded strains MG-104 and MG-105 from MG-103, and MDS-204 and MDS-205 from MDS-203. At each step of the strain constructions, the modification of each target region was verified by PCR using pairs of primers that flanked the endpoints of each target region.

### Bioassay for L-threonine using an *E. coli *threonine auxotroph

The threonine auxotroph MG1655 *thrB*::Tn*5 *[32] was inoculated into 3 ml of LB medium supplemented with kanamycin and grown at 37°C. When the OD_600 _reached 0.8, the threonine auxotrophic cells were harvested by centrifugation at 6,000 g for 10 min, resuspended in 3 ml of fresh M9 minimal medium, and cultivated for 5 h at 37°C to deplete any endogenous threonine available to the cells [33]. The threonine auxotrophic culture was then diluted 1:100 into 3 ml of fresh M9 minimal medium to which 300 μl of the filterate of the culture broth of each threonine producing strains was added. After 24 h of cultivation at 37°C, growth of the threonine auxotroph was measured at OD_600_.

A standard growth curve was established in order to correlate the growth of the auxotroph to various concentrations of threonine in M9 minimal media. Linear regression analysis of the plotted data was considered a least-squares fit (R^2^) of the relationship between threonine supplementation and growth of the auxotroph, and was used as a standard curve when R^2 ^was greater than 0.95. The R^2 ^value was derived from the regression line of the resulting plots (OD_600 _vs. threonine concentration).

### Threonine production by flask fermentation

The threonine-producing strains were grown on LB plates overnight and then transferred to a 250 ml flask containing 50 ml of seed medium [32.5 g glucose, 24.35 g K_2_HPO_4_, 9.5 g KH_2_PO_4_, 15 g yeast extract, 5 g (NH_4_)_2_SO_4_, 1 g MgSO_4_·7H_2_O per liter at pH 7.0]. After growing the culture for 24 h at 37°C, an aliquot (1 ml) of the seed culture was transferred to 50 ml of fermentation medium-1 [2 g yeast extract, 2 g citric acid, 25 g (NH_4_)_2_SO_4_, 7.46 g KH_2_PO_4_, 40 g glucose, 2 g MgSO_4_·7 H_2_O, 5 mg FeSO_4_·7 H_2_O, 5 mg MnSO_4_·4 H_2_O, and 20 g CaCO_3 _per liter at pH 7.2]. The fermentation was run for 24 h at 37°C, with vigorous agitation of the culture on a shaker (300 rpm). After cultivation, the amount of threonine accumulated in the broth was analyzed with the bioassay system using the threonine auxotroph described above.

### Threonine production by batch fermentation

MDS-205 was grown in a 2-liter jar fermentor containing 1.5 liters of fermentation medium-2 [100 g glucose, 10 g (NH_4_)_2_SO_4_, 2 g KH_2_PO_4_, 0.5 g MgSO_4_,·7 H_2_O, 5 mg FeSO_4_·7 H_2_O, 5 mg MnSO_4_·4 H_2_O, and 3 g yeast extract per liter at pH 7.5]. A seed culture was grown for 12 h at 37°C in a 500 ml flask containing 75 ml of seed medium and then inoculated into the 2-liter jar fermentor. During batch phase fermentation, the pH was maintained at 7.5 with NH_4_OH, the temperature at 37°C, the aeration rate at 1 vvm (air volume·working volume^-1^·min^-1^), and the agitation speed at 800 rev/min. After 30 h of fermentation, the concentration of threonine was determined by pre-column derivation with OPA (o-phthaldehyde-thiol) using the method developed by Joseph and Marsden [34] with the following modifications. Threonine was analysed on a Micra NPS ODS-1 (33 mm × 4.6 mm) 1.5-μm column (Eichrom Technologies, IL, USA) in reversed phase with a concentration gradient of sodium acetate buffer. This gradient was formed from two buffers, 100 mM sodium acetate, pH 5.9 (adjusted with 1 M HCl; buffer A) and pure methanol (buffer B), with a flow rate of 0.5 ml/min. The time course of the gradient was as follows: the starting point, buffer A/buffer B (98:2, v/v); 1 min, A/B (85:15, v/v); 5 min, A/B (50:50, v/v); 10 min, A/B (30:70, v/v); 18 min, A/B (2:98, v/v). The retention times and response factors of the threonine were evaluated by injecting known amounts of L-threonine.

### Microarray analysis

Strains MG1655, MDS42, MG-105, and MDS-205 were inoculated from single colonies into 5 ml of seed medium and grown at 37°C overnight. From each overnight culture, 500 μl was used to inoculate 50 ml of fresh fermentation medium-1. These cultures were grown at 37°C, and the cells were harvested at an early log phase corresponding to an OD_600 _of 0.4. Total RNA was extracted using the MasterPure™ RNA Purification Kit (Epicentre Technologies, Madison, WI, USA) from 1 ml of the early log phase culture. cDNA synthesis and labeling were performed as described in the Affymetrix GeneChip *E. coli Antisense *Genome Array Technical Manual [35]. The resulting labeled cDNAs were hybridized to an Affymetrix *E. coli antisense *genome array. Patterns of hybridization were detected with an Affymetrix Genearray scanner 2500 (Affymetrix, Inc., Santa Clara, CA, USA). The raw data were analyzed using Microarray Analysis Suite version 5.0 (Affymetrix). Every *E. coli *open reading frame (ORF) was assayed by a set of perfect match (PM) and mismatch (MM) probe pairs. If the PM probe showed an intensity that was at least 200 U higher than that of the MM probe, the probe pair was considered to be present [35]. An ORF was considered to be present with 95% confidence if neighboring probe pairs within an ORF were present.

## Results

### Construction of L-threonine-overproducing *E. coli *strains and L-threonine production

**Figure 2:**
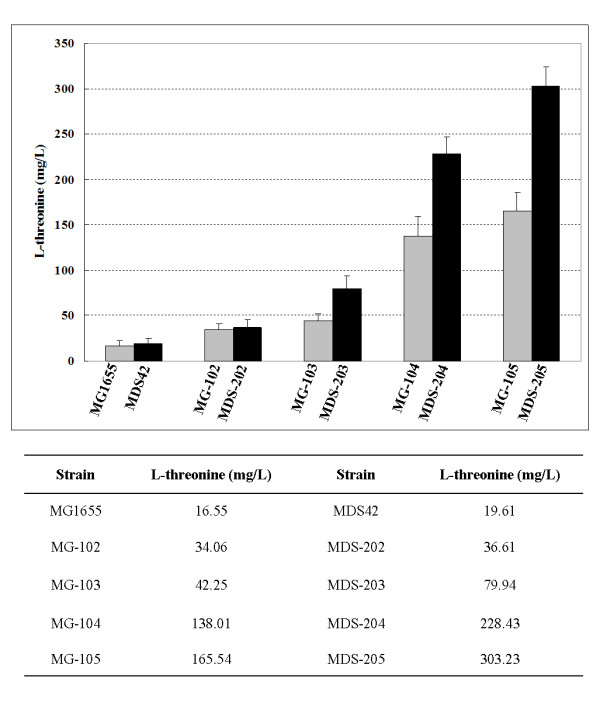
**Production of L-threonine by constructed strains**. The strains were cultivated in 50 ml of fermentation medium-1 at 37°C (refer to Materials and Methods). After 24 h of cultivation, the L-threonine concentration in the culture broth was measured by our bioassay system (refer to Materials and Methods). The data shown are the means and standard deviations for three independent experiments.

The wild-type *E. coli *MG1655 and reduced-genome *E. coli *MDS42 were modified in a stepwise manner to overproduce L-threonine. First, we isolated the threonine operon of *E. coli *strain ATCC 21277, which contains a mutated version of the *thrA *gene (thrA*) that encodes threonine feedback-resistant aspartate kinase I and homoserine dehydrogenase I; a homoserine kinase-encoding gene (*thrB*); and a threonine synthase-encoding gene (*thrC*). The isolated threonine operon, which was expressed under the control of a *Tac *promoter, was then inserted into the genomes of MG1655 and MDS42 to replace their wild-type threonine operons. Second, the *lacI *gene, which encodes the *E. coli *LacI transcriptional repressor, was deleted so that the mutant threonine operon would be expressed constitutively. The resulting strains, MG-102 and MDS-202, produced 34.06 and 36.61 mg/L of L-threonine, respectively (Fig. 2).

Third, the *tdh *gene, which encodes threonine dehydrogenase, was deleted from MG-102 and MDS-202 to prevent the degradation of L-threonine, generating strains MG-103 and MDS-203, which produced 42.25 and 79.94 mg/L of L-threonine, respectively (Fig. 2). Finally, to enhance the export of L-threonine into the culture media and block re-uptake, we sequentially replaced the *tdcC *gene, which encodes the threonine STP importer [36], and the *sstT *gene, which encodes the DctA dicarboxylate (DAACS) importer [37], in MG-103 and MDS-203 with a mutant threonine exporter gene (*rhtA23*). The resulting strains, MG-105 and MDS-205, produced 165.54 and 303.23 mg/L of L-threonine, respectively (Fig. 2). These final strains showed an ~10- and ~15.5-fold increase in L-threonine production, compared to their parental strains, MG1655 and MDS42, respectively (Fig. 2).

When a batch-fermentation was carried out, the final strain MDS-205 produced 40.1 g/L of L-threonine (a yield of 0.401 g threonine/g glucose) after 30 h of fermentation.

### Comparative transcriptome analysis of MG-105 and MDS-205 using DNA microarrays

**Figure 3:**
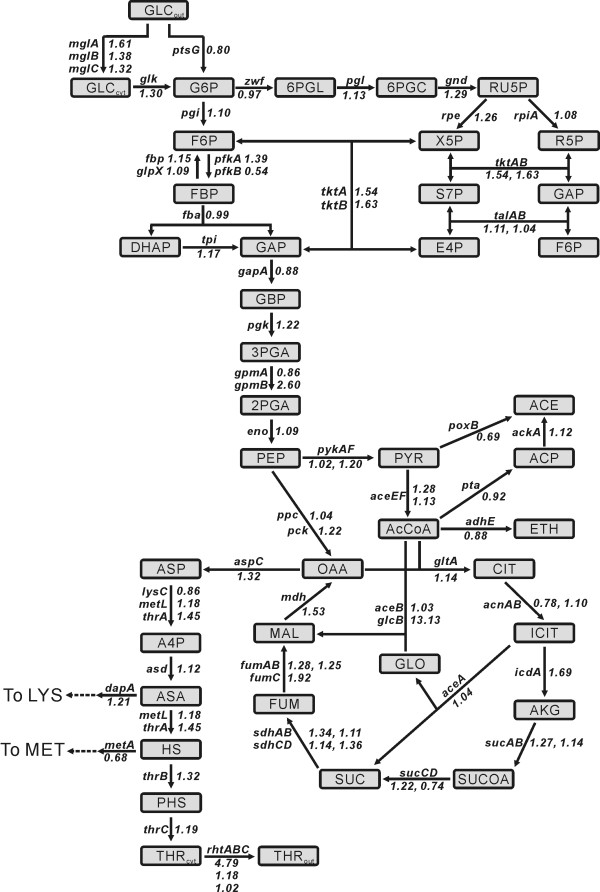
**Relative transcriptional levels of genes related to central metabolism for L-threonine producing strain MG-105 and MDS-205**. Metabolic networks showing the relative transcriptional levels of genes related to the central metalbolism, mixed acid fermentation and L-threonine biosysnthetic pathways. The numbers are the relative ratio of the expression level of MDS-205 as compared to that of MG-105. Metabolite abbreviations: GLC_out_, glucose in medium; GLC_cyt_, glucose in cytoplasm; G6P, glucose-6-phosphate; F6P, fructose-6-phosphate; FBP, fructose-1,6-bisphosphate; DHAP, dihydroxyacetonephosphate; GAP, glyceraldehyde-3-phosphate; GBP, 1,3-bisphosphoglycerate; 3PGA, 3-phosphoglycerate; 2PGA, 2-phosphoglycerate; PEP, phosphoenolpyruvate; PYR, pyruvate; AcCoA, acetyl-CoA; ACP, acetyl-phosphate; ACE, acetate; ETH, ethanol; OAA, oxaloacetate; CIT, citrate; ICIT, isocitrate; AKG, a-ketoglutarate; SUCOA, succinyl-CoA; SUC, succinate; FUM, fumarate; MAL, malate; ASP, aspartate; A4P, aspartyl-4-phosphate; ASA, aspartate semialdehyde; HS, homoserine; PHS, homoserine phosphate; THR_out_, L-threonine in medium; THR_cyt_, L-threonine in cytoplasm; 6PGL, gluconolactone-6-phosphate; 6PGC, 6-phosphogluconate; RU5P, ribulose-5-phosphate; X5P, xylurose-5-phosphate; R5P, ribose-5-phosphate; S7P, sedoheptulose-7-phosphate; E4P, erythrose-4-phosphate.

To understand the difference in global gene expression levels in MG-105 and MDS-205, a transcriptional profiling experiment was performed. One hundred genes (2.7% of the total genes expressed) in MDS-205 cells were differentially expressed by more than 2-fold compared to MG-105 cells (63 up-regulated and 37 down-regulated) (Additional file 2). Genome-scale gene expression analysis of MG-105 and MDS-205 revealed that the most of the genes related to the central metabolism and L-threonine biosynthesis were up-regulated in MDS-205. The expression ratios of genes related to central metabolic pathways and L-threonine biosynthesis are shown in Fig. 3. The expression levels of the *ptsG *gene involved in the glucose PTS-system was down-regulated by 0.80-fold in MDS-205, whereas genes involved in the non-PTS glucose uptake system, *mglABC *and *glk*, were up-regulated by 1,61-, 1.38-, 1.32- and 1.30-fold, respectively. In addition, the *tktAB *genes involved in the pentose phosphate shunt were up-regulated by 1.54-, and 1.63-fold in MDS-205, respectively. And the *pck *gene involved in the carboxylation of phosphoenolpyruvate was up-regulated by 1.22-fold in MDS-205. Meanwhile genes involved in the mixed acid fermentation (*poxB*, *pta*, and *adhE*) were down-regulated in MDS-205. The *aspC *gene, which products directs oxaloacetate toward L-threonine biosyntheis, the *glcB *gene involved in the glyoxylate-shunt, and genes involved in TCA cycle were also up-regulated in MDS-205. The *thrABC *genes involved in L-threonine biosynthesis and the *rhtA *gene responsible for the L-threonine export were up-regulated by 1.45-, 1.32-, 1.19-, and 4.79-fold, respectively, in MDS-205.

## Discussion

The restructuring of microbial genomes by eliminating genes that are unnecessary for a cellular metabolism has received special attention as an important strategy for industrial strain improvement [1-5]. Recently, scientists reported on the advantages of the reduced-genome *E. coli *strain MDS42 and its applications for bioindustry [5,38]. The MDS42 genome is 14.3% smaller than that of the wild-type strain MG1655 and has ~700 fewer genes.

In this study, we compared the metabolic efficiency of L-threonine production in the wild-type *E. coli *strain MG1655 and reduced-genome *E. coli *strain MDS42 by introducing into each of these strains a series of genetic modifications that altered L-threonine production, degradation, export into the media, and re-uptake from the media. These modifications gave rise finally to MG-105 (from MG1655) and MDS-205 (from MDS42). Although both MG1655 and MDS42 went through the same modifications, the threonine production of MDS-205 strain was an ~2-fold greater than that of the MG-105 strain.

Genome-scale gene expression analysis of MG-105 and MDS-205 revealed that the most of the genes involved in the central metabolism and L-threonine biosynthesis were up-regulated in MDS-205. Among the up-regulated genes, the expression level of the *rhtA *threonine exporter gene was increased by 4.79-fold in MDS-205. This result indicates that the maximization of L-threonine export combined with deletion of the *tdcC *and *sstT *gene involved in the re-uptake of L-threonine across the membrane is one of the important steps for the mass production of L-threonine from *E. coli*. An additional advantage of the overexpression of the *rhtA *gene is that it increases the tolerance of *E. coli *to L-threonine by an ~3-fold [31], which helps the *E. coli *to withstand high concentrations of L-threonine in the media. This adaptation also might contribute to the higher L-threonine production.

The up-regulation of the ATP-dependent glucose transport and phosphorylation system (*mglABC *and *glk *genes) and down-regulation of the PTS system (*ptsG *gene) in MDS-205 may increase the availability of the phosphoenolpyruvate (PEP) which is a precursor of oxaloacetate (OAA) and aspartate. This result is consistent with the prediction based on flux balance analyses of gene knock-outs in an *E. coli *metabolic model that the replacement of PTS activity by an ATP-dependent glucose transport system should increase aspartate-family amino acids [39,40]. In addition, down-regulation of *poxB*, *pta*, and *adhE *genes, which are directing PEP to acetate and ethanol, may reduce the PEP flux to acetate and ethanol and further increase the PEP availability.

It is also reported that the up-regulation of *pck *gene involved in the carboxylation of PEP to OAA leads to increased cellular growth and biomolecular production, since the *ppc *reaction releases an inorganic phosphate, in contrast to the *pck *reaction, which produces a high-energy ATP [41-44]. The effect of the pentose phosphate shunt and glyoxylate bypass on the glycolytic flux to L-threonine production also has been reported [45]. Therefore, the up-regulation of the *pck *gene involved in the PEP carboxylation and *tktAB *genes involved in the pentose phosphate shunt and the increased expression of the genes involved in TCA cycle and the glyoxylate bypass (*mdh*, *fumABC*, *sdhABCD*, and *glcB *genes) may increase the OAA synthesis in MDS-205, resulted in higher production of L-threonine in MDS-205 compared to MG-105. The increased PEP and OAA level of MDS-205 by increasing the non-PTS glucose uptake system, PEP carboxylation, the pentose phosphate pathway, and the glyoxylate shunt, and reducing the mixed acid fermentation may resulted in increased production of L-threonine.

**Figure 4:**
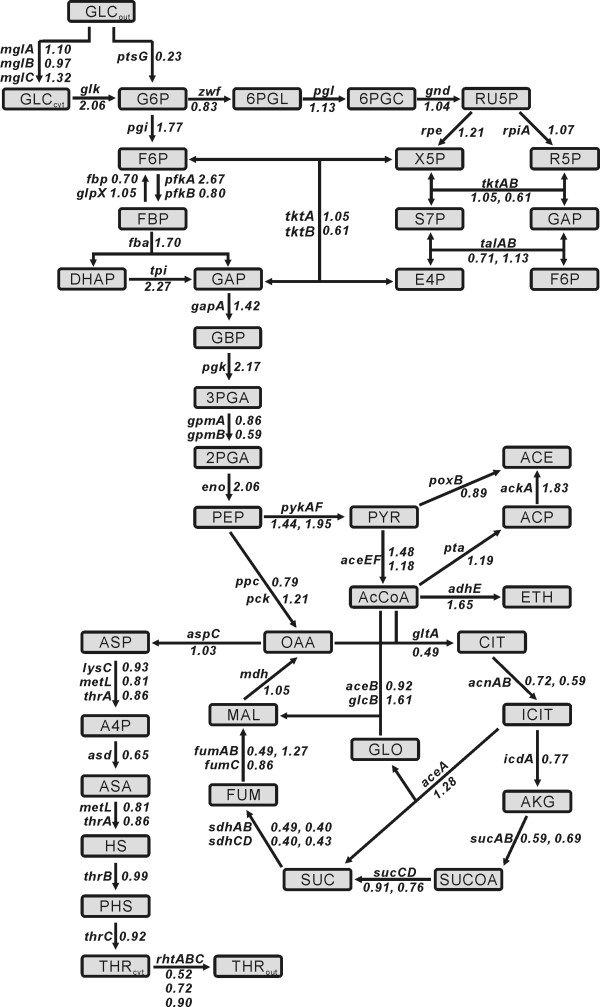
**Relative transcriptional levels of genes related to central metabolism for L-parental strain MG1655 and MDS42**. Metabolic networks showing the relative transcriptional levels of genes related to the central metalbolism, mixed acid fermentation and L-threonine biosysnthetic pathways. The numbers are the relative ratio of the expression level of MDS42 as compared to that of MG1655. Metabolite abbreviations: GLC_out_, glucose in medium; GLC_cyt_, glucose in cytoplasm; G6P, glucose-6-phosphate; F6P, fructose-6-phosphate; FBP, fructose-1,6-bisphosphate; DHAP, dihydroxyacetonephosphate; GAP, glyceraldehyde-3-phosphate; GBP, 1,3-bisphosphoglycerate; 3PGA, 3-phosphoglycerate; 2PGA, 2-phosphoglycerate; PEP, phosphoenolpyruvate; PYR, pyruvate; AcCoA, acetyl-CoA; ACP, acetyl-phosphate; ACE, acetate; ETH, ethanol; OAA, oxaloacetate; CIT, citrate; ICIT, isocitrate; AKG, a-ketoglutarate; SUCOA, succinyl-CoA; SUC, succinate; FUM, fumarate; MAL, malate; ASP, aspartate; A4P, aspartyl-4-phosphate; ASA, aspartate semialdehyde; HS, homoserine; PHS, homoserine phosphate; THR_out_, L-threonine in medium; THR_cyt_, L-threonine in cytoplasm; 6PGL, gluconolactone-6-phosphate; 6PGC, 6-phosphogluconate; RU5P, ribulose-5-phosphate; X5P, xylurose-5-phosphate; R5P, ribose-5-phosphate; S7P, sedoheptulose-7-phosphate; E4P, erythrose-4-phosphate.

The increased productivity of L-threonine in MDS42 might also result from a decrease in the metabolic burden due to the genome reduction. It is well known that the maintenance and expression of unnecessary genes, such as plasmid DNA, impose an uncharacterized metabolic burden on the bacterial host [46]. The metabolic burden could arise due to the extra biosynthetic demands for synthesis and expression of unnecessary genes [47,48], or the perturbation of the *E. coli *host regulatory system affecting central metabolic pathways [46,49]. The genome-scale gene expression analysis of the wild-type MG1655 and the MDS42 revealed that the genes involved in the non-PTS glucose uptake system and glycolysis were up-regulated in MDS42 compared to MG1655, whereas the genes involved in the glucose PTS-system and TCA cycle were down-regulated in MDS42 compared to MG1655 (Fig 4). It seems that the elimination of unnecessary genes from the *E. coli *genome might lead to more efficient cellular metabolism and an improved substrate yield coefficient, resulting in nutrient and energy saving of cells without physiological compromise. Researchers reported that the elimination of unnecessary genes actually improves *E. coli *robustness and enhances carbon metabolism [5,38,50]. In fact, genes involved in mixed acid fermentation were up-regulated in MDS42 compared to MG1655 (Fig 4), leading to the acetate accumulation which indicates a carbon-overflow metabolism [13]. Therefore, the increased production of L-threonine in MDS-205 might be resulted from the redirection of the overflowed carbon metabolism in MDS42 caused by elimination of unnecessary genes from the *E. coli *genome into the production of L-threonine.

## Conclusion

In this study, we report the results of a reengineering of MDS42 to increase production of the amino acid L-threonine, which is essential for growth and maintenance of commercial livestock [25]. Even though a series of systematic experiments is needed for better understanding of the mechanism underlying the higher L-threonine production in the reduced-genome *E. coli *MDS42, our results described herein clearly indicate that MDS42 can serve as an efficient host strain for the production of other useful biomaterials.

## Competing interests

The authors declare that they have no competing interests.

## Authors' contributions

JL and BS carried out the construction and analysis of the threonine producing strains. BY and JK participated in the fermentation study. FB participated in the genome-scale transcriptional analysis. MK, and SK helped to draft the manuscript. All authors read and approved the final manuscript.

## Supplementary Material

Additional file 1 **Table S1. List of all primers used in this study**. Click here for file

Additional file 2 **Table S2. List of all genes that were differentially expressed in MDS-205, relative to MG-105.** Click here for file
