# Supplementary material for: Metabolic engineering of a reduced-genome strain of Escherichia coli for L-threonine production
Source: Microb Cell Fact. 2009 Jan 7;8:2. doi: 10.1186/1475-2859-8-2 (PMC2634754; doi:10.1186/1475-2859-8-2)
Supplement: Additional file 1 — Table S1. List of all primers used in this study. [file 1475-2859-8-2-S1.doc]

**Table S1. List of all primers used in this study**

| **Primer name** | | **Seqeunce (5’*→*3’)** |
| --- | --- | --- |
| thrABC operon | *P-f1* | ata caa caa ccg gcg aaa ag |
|  | *P-r1* | cga cgc tca tat tgg cac t |
|  | *P-f2* | GAT GCA ACG GCA GAC CAA CAT CAA CTG CAA GCT TTA CGC GAA CGA GCC ATG CTG GCT AAT TTC GCA GTT C |
|  | *P-r2* | GTG ACT ACA TCT CCG AGC AAT GCA CCA TCA ACA GGT GTC ACC GCC GCC CC cga cgc tca tat tgg cac t |
| tdcC | *P-f1* | CGG CTA TGT GTT CCG CTA TT |
|  | *P-r1* | CCT CAA ATG TGA TTC AAA TAA GTC C |
|  | *P-f2* | ATT AAA ACA GTT TGT ATA CGA TGT TCA GGA TGG TCA GCA GAC CAA TCA CG GAT CTG GCT TAT CGA AAT TAA T |
|  | *P-r2* | CTT ACG TGC CGA TCA ACG TCT CAT TTT CGC CAA TAG GGA TAA CAG GGT AA CCT CAA ATG TGA TTC AAA TAA GTC C |
| sstT | *P-f1* | CGG CTA TGT GTT CCG CTA TT |
|  | *P-r1* | CCT CAA ATG TGA TTC AAA TAA GTC C |
|  | *P-f2* | TTG GCT TTA TTT TTA TGT CAA AGA AAT GTA ACC ATT AAG TTT CAA AAT AT GAT CTG GCT TAT CGA AAT TAA T |
|  | *P-r2* | GTT TAA AGT TGA GAA AAC CCC TTC CGC CGT AGA CGA AAG GGG TTA AAC AA CCT CAA ATG TGA TTC AAA TAA GTC C |
