# Supplementary material for: Metabolic engineering of a reduced-genome strain of Escherichia coli for L-threonine production
Source: Microb Cell Fact. 2009 Jan 7;8:2. doi: 10.1186/1475-2859-8-2 (PMC2634754; doi:10.1186/1475-2859-8-2)
Supplement: Additional file 2 — Table S2. List of all genes that were differentially expressed in MDS-205, relative to MG-105. [file 1475-2859-8-2-S2.doc]

**Table S2. List of all genes that were differentially expressed in MDS-205**, relative to MG-105

| **Gene**  **name** | **Function^a^** | **Log_2_**  **fold difference^b^** |
| --- | --- | --- |
| *rspB* | enzyme; global regulatory functions | 6.04 |
| *rspA* | phenotype; global regulatory functions | 5.92 |
| *b1788* | ORF; unknown | 3.20 |
| *dcp* | enzyme; degradation of proteins, peptides, glyco-proteins | 2.91 |
| *dsrA* | RNA; regulatory RNA for genes encoding surface polysaccharides & antigens; regulates transcriptional silencing by H-NS protein and enhances translation of RpoS | 2.79 |
| *yihU* | putative enzyme; not classified | 2.28 |
| *xdhB* | putative enzyme; not classified | 2.18 |
| *ychM* | ORF; unknown | 2.06 |
| *cynX* | transport; transport of small molecules: anions | 2.00 |
| *csgE* | structural component; surface structures | 1.86 |
| *amtB* | putative transport related to central intermediary metabolism | 1.82 |
| *ycdN_2* | putative carrier; not classified | 1.80 |
| *csgF* | structural component; Surface structures | 1.77 |
| *cysC* | enzyme; Central intermediary metabolism: Sulfur metabolism | 1.68 |
| *molR_2* | regulator; Biosynthesis of cofactors, carriers: Molybdopterin | 1.68 |
| *agaD* | enzyme; Central intermediary metabolism: Amino sugars | 1.63 |
| *agaC* | enzyme; Central intermediary metabolism: Amino sugars | 1.61 |
| *yjbE* | ORF; Unknown | 1.56 |
| *yjfM* | ORF; Unknown | 1.55 |
| *phoR* | enzyme; Global regulatory functions | 1.54 |
| *yhjO* | putative enzyme; Not classified | 1.45 |
| *yohH* | ORF; Unknown | 1.45 |
| *yjjJ* | ORF; Unknown | 1.45 |
| *ydcH* | ORF; Unknown | 1.37 |
| *dsdX* | transport; Not classified | 1.37 |
| *yjfO* | ORF; Unknown | 1.37 |
| *b3814* | ORF; Unknown | 1.36 |
| *yedR* | ORF; Unknown | 1.32 |
| *ydeI* | ORF; Unknown | 1.32 |
| *gabD* | enzyme; Central intermediary metabolism | 1.31 |
| *b2670* | ORF; Unknown | 1.29 |
| *bglF* | enzyme; Transport of small molecules: Carbohydrates, organic acids, alcohols | 1.27 |
| *yohC* | ORF; Unknown | 1.27 |
| *ydjO* | ORF; Unknown | 1.27 |
| *eutE* | putative enzyme; Degradation of small molecules: Amines | 1.27 |
| *molR_1* | regulator; Biosynthesis of cofactors, carriers: Molybdate | 1.19 |
| *yohF* | putative enzyme; Not classified | 1.18 |
| *ssuC* | putative transport; Not classified | 1.16 |
| *b2772* | ORF; Unknown | 1.13 |
| *yodA* | ORF; Unknown | 1.12 |
| *yehW* | putative transport; Not classified | 1.12 |
| *yncC* | ORF; Unknown | 1.12 |
| *ygcS* | putative transport; Not classified | 1.11 |
| *csgD* | putative regulator; Not classified | 1.10 |
| *ycbQ* | putative structure; Not classified | 1.10 |
| *yncH* | ORF; Unknown | 1.09 |
| *sbp* | transport; Transport of small molecules: Anions | 1.07 |
| *ccmF* | putative enzyme; Energy metabolism, carbon: Electron transport | 1.07 |
| *phoB* | regulator; Global regulatory functions | 1.07 |
| *yeaV* | putative transport; Not classified | 1.07 |
| *ygcJ* | ORF; Unknown | 1.05 |
| *ygcP* | putative regulator; Not classified | 1.05 |
| *prpR* | regulator; Not classified | 1.04 |
| *ansP* | transport; Transport of small molecules: Amino acids, amines | 1.04 |
| *cusA* | putative transport; Not classified | 1.03 |
| *pheP* | transport; Transport of small molecules: Amino acids, amines | 1.02 |
| *yraK* | ORF; Not classified | 1.02 |
| *ygbK* | ORF; Unknown | 1.02 |
| *yceO* | ORF; Unknown | 1.02 |
| *ycdB* | ORF; Unknown | 1.00 |
| *yciE* | ORF; Unknown | 1.00 |
| *cusS* | putative regulator; Not classified | 1.00 |
| *phnK* | transport; Central intermediary metabolism: Phosphorus compounds | 1.00 |
| *ydhV* | ORF; Unknown | -1.02 |
| *yabQ* | ORF; Unknown | -1.03 |
| *yjdI* | ORF; Unknown | -1.03 |
| *mdoH* | enzyme; Osmotic adaptation | -1.04 |
| *yhbW* | putative enzyme; Not classified | -1.04 |
| *glnG* | regulator; Amino acid biosynthesis: Glutamine | -1.10 |
| *ttdA* | enzyme; Energy metabolism, carbon: Fermentation | -1.10 |
| *yciK* | putative enzyme; Not classified | -1.12 |
| *yjcO* | ORF; Unknown | -1.13 |
| *dcuB* | transport; Transport of small molecules: Carbohydrates, organic acids, alcohols | -1.13 |
| *nanA* | enzyme; Surface polysaccharides & antigens | -1.14 |
| *ygfS* | putative enzyme; Not classified | -1.16 |
| *yiiP* | putative transport; Not classified | -1.18 |
| *yojI* | putative transport; Not classified | -1.19 |
| *yohJ* | ORF; Unknown | -1.19 |
| *nanT* | transport; Murein sacculus, peptidoglycan | -1.21 |
| *tnaA* | enzyme; Degradation of small molecules: Amino acids | -1.21 |
| *yraM* | putative enzyme; Not classified | -1.31 |
| *tnaL* | leader; Degradation of small molecules: Amino acids | -1.36 |
| *gpmB* | enzyme; Energy metabolism, carbon: Glycolysis | -1.38 |
| *yghW* | ORF; Unknown | -1.38 |
| *tpr* | factor; Basic proteins: synthesis, modification | -1.48 |
| *csgC* | putative curli production protein | -1.52 |
| *hybF* | regulator; Energy metabolism, carbon: Anaerobic respiration | -1.55 |
| *yigE* | ORF; Unknown | -1.71 |
| *dcd* | enzyme; 2'-Deoxyribonucleotide metabolism | -1.78 |
| *yhiH* | putative transport; Not classified | -1.82 |
| *proL* | RNA; tRNA | -1.96 |
| *yeaU* | putative enzyme; Not classified | -1.97 |
| *ybiF* | putative membrane component; Not classified | -2.26 |
| *yibD* | putative regulator; Not classified | -2.36 |
| *rhaT* | transport; Transport of small molecules: Carbohydrates, organic acids, alcohols | -2.48 |
| *argG* | enzyme; Amino acid biosynthesis: Arginine | -2.76 |
| *ymdA* | ORF; Unknown | -3.23 |
| *yecR* | ORF; Unknown | -3.46 |
| *ompT* | enzyme; Outer membrane constituents | -5.07 |

^a^ From the EcoCyc database ([http://www.ecocyc.org](http://www.ecocyc.org/))

^b^ log_2_ fold difference in gene expression between MDS-205 and MG-105, both grown in fermentation medium-1 at 37˚C (refer to Materials and Methods)
